# Younger age of patients with myocardial infarction is associated with a higher number of relatives with a history of premature atherosclerosis

**DOI:** 10.1186/s12872-020-01677-w

**Published:** 2020-09-11

**Authors:** Michał Ambroziak, Katarzyna Niewczas-Wieprzowska, Agnieszka Maicka, Andrzej Budaj

**Affiliations:** Department of Cardiology, Centre of Postgraduate Medical Education, Grochowski Hospital, Grenadierów 51/59, 04-730 Warsaw, Poland

**Keywords:** Myocardial infarction at a young age, Premature coronary artery disease, CVD family history

## Abstract

**Background:**

Premature coronary artery disease is one of the most pressing global issues in modern cardiology. The aim of the study was to investigate the role of family history of premature cardiovascular disease (CVD) in patients aged < 50 years with myocardial infarction (MI) compared to that in patients aged ≥50 years with MI and to that in young people without MI (no-MI < 50).

**Methods:**

The studied group (MI < 50) consisted of 240 patients aged 26–49 years with MI. The control groups consisted of 240 patients (MI ≥ 50) with MI aged 50–92 years and 240 healthy people aged 30–49 years without a history of MI (no-MI < 50).

**Results:**

There were statistically significant differences between the MI < 50 and MI ≥ 50 and no-MI < 50 groups regarding the family history of premature MI/ischaemic stroke and the percentage of patients with ≥2 relatives affected (10.8, 2.9, and 3.7%, respectively; *p* < 0.0001). There was a statistically significant difference in the patient age at the first MI occurrence among patients without a family history of premature CVD, those with 1 affected relative, and those with ≥2 affected first-degree relatives (56.6, 48.6 and 41.8 years, respectively) as well as those with affected first- and second-degree relatives (56.5, 50.7 and 47.0 years, respectively).

**Conclusions:**

A younger age of patients with myocardial infarction is associated with a higher number of relatives with a history of premature MI/ischaemic stroke. Thus, the family history of premature atherosclerosis involving not only first- but also second-degree relatives seems to be a valuable factor in CVD risk evaluation in young people.

**Graphical Abstract:**

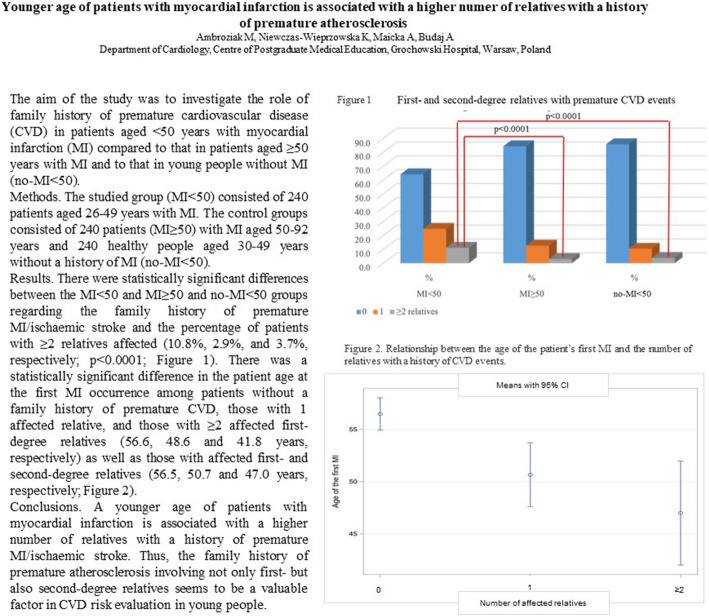

## Background

Coronary artery disease (CAD), according to a report of the American Heart Association, remains the leading cause of cardiovascular disease (CVD) deaths [1]. Regarding this, premature CAD seems to be one of the most pressing global issues in this area.

Data regarding the prevalence of myocardial infarction (MI) in young people differ according to assumptive cut-off age and study population. The percentage of patients aged < 35 years who underwent cardiac catheterization due to MI was determined to be 2% [2]. Recently published data reported that 10% of patients with MI, ST-elevation MI (STEMI), non-ST-elevation MI (NSTEMI), and unstable angina (UA) were ≤ 40 years of age [3]. Patients aged < 40 years represented 1.2% of all patients with MI in a Polish study [4]. When a cut-off age of 45 was established, the percentage of patients increased to 3.2% [5]. The percentage of adults aged < 55 years with MI within participants of the Global Registry of Acute Coronary Events (GRACE) was 23 [6]. In one of the recently published meta-analyses, the cut-off age for a young age of acute coronary syndrome (ACS) was established at 50 [7].

Family history appears to be one of the most important and significant risk factors in young patients with MI. It covers inherited genetic as well as environmental risk factors (diet, lifestyle, smoking). A recently published Polish study (the MAGNETIC Project) revealed that young adults with a family history of premature CAD presented unfavourable dietary patterns, which suggested a possible continuity of familial lifestyle across generations [8].

A family history of premature CV events is defined as MI or ischaemic stroke in first-degree relatives at age < 55 years in men and < 65 years in women [9, 10]. The INTERHEART study indicated that parental history of CAD was a risk factor independent of environmental, cultural, behavioural, classic, and genetic conditions [11]. Furthermore, the age of onset of disease in parents and whether one or both parents are affected are valuable information and provide an assessment of individual risk of MI. Nevertheless, data regarding the role of a history of premature CV events in family members other than parents are scarce.

The aim of the study was to analyse risk factors for MI at a young age, particularly the role of a family history of CVD. Regarding various of cut-off ages for young MI among the literature, we used the age of 50 years in this study. We investigated the family history of premature MI/ischaemic stroke in patients with MI at age < 50 compared to that in patients with MI at age ≥ 50 and to that in young people without MI (no-MI < 50). We assessed correlations between the number of relatives affected and the age of MI patients, including not only parents but also other family members, such as siblings, grandmothers, grandfathers, children and siblings of parents.

## Methods

### Patients

The investigated population included 720 persons, some of whom were participants in the previously published study [12].

The studied group consisted of 240 young patients aged < 50 years (mean age 43.5, SD ±5.0; range 26–49) admitted to the Department of Cardiology, Centre of Postgraduate Medical Education, Grochowski Hospital (Warsaw) with first-episode MI diagnosed based on ST changes in ECG, including STEMI (ST-elevation MI) as well as NSTEMI (non-ST-elevation MI), serum troponin levels and clinical manifestation. The group (MI < 50 group) consisted of 188 men and 52 women (78.3 and 21.7%, respectively).

The data from the studied group were compared to those of the control group (MI ≥ 50 group), including 240 patients admitted to our department due to the first-episode MI, aged ≥50 years, range 50–92 (mean 65.9 years, SD ±12.6), including 152 men and 88 women (63.3 and 36.7%, respectively).

All patients with MI (groups MI < 50 and MI ≥ 50) had coronary artery atherosclerosis confirmed by angiography. In both groups of MI patients, the exclusion criteria were previous MI, MI type 2 without atherosclerotic changes in coronary arteries and a lack of consent for participation in the study.

The other control group consisted of young people without MI, aged, similar to the studied group < 50 years (mean 43.2 years, SD ±5.0, range from 30 to 49 years), without a history of CAD. This group (no-MI < 50) included 137 men and 103 women (57.1 and 42.9%, respectively). These participants were recruited from the Regional Blood Centre and a general practitioner outpatient clinic. The exclusion criteria for the healthy control group were MI or angiographic features of CAD and a lack of consent for participation in the study.

We collected detailed information regarding family history of premature CVD (MI/ischaemic stroke in men aged < 55 and in women aged< 65), body mass index (BMI), smoking, hypertension, diabetes mellitus (DM) and depression from all participants of the study (together 720 persons). Data regarding family history were collected during the interview with a clinician, a medical doctor. The detailed questions were asked, including the specific diagnosis of heart disease in a family member, the exact patient age of an event occurrence and specific information regarding kinship. In case of doubt regarding the family history, the patient had an opportunity to contact the family member to obtain precise information, if available. If the information provided by the patient was not clear or certain, it was not included in the analysis.

Data regarding risk factors and comorbidities were collected during the interview with a clinician, a medical doctor, at the first meeting at baseline. When there were doubts regarding previous diagnoses, the patient was asked to provide medical documentation.

Hypertension was defined, according to ESH/ESC (European Society of Hypertension, European Society of Cardiology) guidelines, as values ≥140 mmHg for systolic blood pressure (SBP) and/or ≥ 90 mmHg for diastolic blood pressure (DBP), based on repeated blood pressure measurements, medical history and ongoing blood-pressure-lowering treatment [13]. DM was assessed according to WHO and ADA (American Diabetes Association) guidelines based on at least two glucose measurements of fasting plasma glucose ≥126 mg/dl or ≥ 200 mg/dl in oral glucose tolerance test measurements, based on medical history and hypoglycaemic ongoing treatment [14]. Smoking refers to both current smoking and former smoking. Occasional smoking in the distant past (> 10 years until baseline) or a total number of cigarettes smoked per life less than 100 were excluded from this category, and these patients were treated as non-smokers.

The investigation conforms to the principles outlined in the Declaration of Helsinki. The study protocol was approved by the Ethical Committee of the Centre of Postgraduate Medical Education. The participants gave written informed consent for participation in the study.

### Biochemical analyses

The blood for all biochemical analyses, including glucose, total cholesterol, HDL and LDL cholesterol and triglyceride (TG) plasma concentrations, was taken in the early morning after admission to the hospital. Analyses were determined from fasting blood samples by standard enzymatic methods using COBAS INTEGRA 800 reagents and equipment (Roche Diagnostics Gmbh, Manheim, GE).

### Statistical analyses

Continuous data are presented as arithmetic means and SDs for normally distributed variables and medians and interquartile ranges (25–75th) for skewed distributions. Differences between means were examined with t-tests. Before using the analysis logarithmic transformations were used for triglicerides and glucose. The differences in binary proportions between groups were analysed using the chi2 test of independence or Fisher’s exact test. Cochran-Mantel-Haenszel modified ridit scores for categorical variables with > 2 categories were used. All hypotheses were two-tailed with a type I error rate of 0.05. All calculations were performed with SAS software version 9.4 (SAS Institute Inc., Cary, NC, USA).

## Results

### Clinical and metabolic characteristics of the studied groups

There was a statistically significant higher prevalence of smoking (86.4% vs 64.3%, *p* < 0.001), BMI (28.6 kg/m^2^ vs 27.6 kg/m^2^, *p* = 0.012), total cholesterol (210.0 mg% vs 197.7 mg%, *p* = 0.006) and TG levels (145.5 mg% vs 1113.0 mg%, *p* < 0.001) in the MI < 50 group than in the MI ≥ 50 group. Prevalence of hypertension (55.2% vs 68.1%, p < 0.001), DM (15.9% vs 35.7%, p < 0.001), HDL cholesterol (41.5 mg% vs 47.6 mg%, p < 0.001) and fasting glucose (99.0 mg% vs 105.4 mg%, *p* = 0.002) levels were significantly lower in the MI < 50 group than in the MI ≥ 50 group (Table 1).

**Table 1:** Clinical characteristics of the studied groups: patients with MI aged < 50 years (MI < 50), patients with MI aged ≥50 years (MI ≥ 50) and young people without history of MI/CAD aged < 50 years (no-MI < 50); ns – not significant, ^a^ - data for TG and glucose showed as median and 25-75th quartile (variables with non-normal distribution)

	MI < 50 *n* = 240	MI ≥50 n = 240	no-MI < 50 n = 240	*p* value	p value
	a	b	c	a vs b	a vs c
BMI (mean ± SD) kg/m2	28.6 ± 4.4	27.6 ± 4.0	27.0 ± 4.3	0.012	< 0.001
Smoking n (%)	203 (86.4)	151 (64.3)	103 (43.1)	< 0.001	< 0.001
Hypertension n (%)	132 (55.2)	162 (68.1)	70 (29.2)	< 0.001	< 0.001
Diabetes mellitus n (%)	36 (15.9)	80 (34.5)	2 (0.8)	< 0.001	< 0.001
Depression n (%)	20 (8.4)	20 (8.4)	8 (3.3)	ns	0.018
Total cholesterol (mean ± SD) mg/dL	210.0 ± 41.5	197.7 ± 50.4	203.5 ± 45.4	0.006	ns
LDL (mean ± SD) m/dL	132.4 ± 39.5	124.5 ± 44.6	123.3 ± 36.5	ns	0.049
HDL (mean ± SD) mg/dL	41.5 ± 12.8	46.7 ± 13.9	54.1 ± 20.3	< 0.001	< 0.001
TG ^a^ (median, [25-75th] quartile) mg/dl	145.5 [104.0–219.0]	113.0 [79.0–155.0]	120.0 [80.0–150.0]	< 0.001	< 0.001
Glucose ^a^ (median, [25-75th] quartile) mg/dl	99.0 [92.5–111.0]	105 [91.0–127.0]	93.0 [84.0–97.0]	0.002	< 0.001

Statistically significant differences between the MI < 50 group and the no-MI < 50 group included smoking (86.4% vs 43.1%, respectively, *p* < 0.001), BMI (28.6 kg/m^2^ vs 27.0 kg/m^2^, *p* < 0.001), prevalence of hypertension (55.2% vs 29.2%, *p* < 0.001), DM (15.9% vs 0.8%, *p* < 0.001) and depression (8.4% vs 3.3%, *p* = 0.018) as well as LDL cholesterol (132.4 mg% vs 123.3 mg%, *p* = 0.049), HDL (41.5 mg% vs 54.1 mg%, p < 0.001), TG (145.5 mg% vs 120.0 mg%, p < 0.001) and glucose (99.0 mg% vs 93.0 mg%, p < 0.001) levels.

There were 16.7, 26.2 and 0.8% participants on statin treatment at baseline in the MI < 50, MI ≥ 50 and no-MI < 50 groups, respectively. There were no individuals using or addicted to cocaine, HIV infected or affected by other severe communicable diseases neither within the study group of MI < 50 nor both control groups of MI ≥ 50 and no-MI < 50 individuals.

### Socioeconomic characteristics of the studied groups

There were significant differences between the MI < 50 group and the MI ≥ 50 group in the level of education (percentage of people with primary education 5.7% vs 15.2% respectively, *p* = 0.001), the type of job (blue-collar 48.4% vs 41.3%, respectively, *p* < 0.001) and marital status (percentage of single people: 18.5% vs 8.68% respectively, *p* < 0.001); Table 2.

**Table 2:** Socio-economic status of the studied groups: patients with MI aged < 50 years (MI < 50), patients with MI aged ≥50 years (MI ≥50) and young people without history of MI/CAD aged < 50 years (no-MI < 50); ns – not significant

	MI < 50 n = 240	MI ≥50 n = 240	no-MI < 50 n = 240	p value	p value
	a	b	c	a vs b	a vs c
Education level					
primary	13 (5.7)	35 (15.2)	5 (2.1)	0.001	< 0.001
vocation	75 (32.7)	51 (22.2)	28 (11.9)		
secondary	90 (39.3)	102 (44.3)	104 (44.3)		
high	51 (22.3)	42 (18.3)	98 (41.7)		
Type of job					
unemployed	7 (3.2)	4 (1.8)	1 (0.4)	< 0.001	< 0.001
blue collar	107 (48.4)	93 (41.3)	83 (38.1)		
white collar	85 (38.5)	69 (30.7)	131 (60.1)		
pensioner	22 (9.9)	59 (26.2)	3 (1.4)		
Marriage status					
single	42 (18.5)	20 (8.6)	39 (16.7)	< 0.001	ns
married	166 (73.1)	151 (64.8)	179 (76.8)		
divorced	9 (4.0)	9 (3.9)	12 (5.1)		
widowed	10 (4.4)	53 (22.7)	3 (1.3)		

Comparing the MI < 50 group and the no-MI < 50 group, there were statistically significant differences in the level of education (percentage of people with university degree 22.3% vs 41.7%, respectively, *p* < 0.001) and the type of job (white-collar 38.5% vs 60.1%, respectively, p < 0.001) but not in marital status.

### Family history

There were statistically significant differences among the MI < 50, MI ≥ 50, and no-MI < 50 groups in the presence of a family history of premature CVD in the first-degree relatives: 32.9% vs 9.6% (*p* < 0.0001) and 32.9% vs 11.7% (p < 0.0001), respectively (Table 3). There were also statistically significant differences among the MI < 50, MI ≥ 50 and no-MI < 50 groups in the presence of a family history of premature CVD age involving the first- and second-degree relatives: 35.9% in the MI < 50 group vs 15.6% in the MI ≥50 group (*p* < 0.0001) and 14.2% in the no-MI < 50 group (*p* < 0.0001). Moreover, there were statistically significant differences between the studied groups in the family history of CVD events at every age within family members (the first- and the second-degree relatives): 65.4% in the MI < 50 group vs 47.6% in the MI ≥50 group (p < 0.0001) and 41.7% in the no-MI < 50 group (*p* < 0.0001).

**Table 3:** Family history of premature CVD events (MI/ischemic stroke in men aged < 55 years and in women aged < 65 years) in the studied groups: patients with MI aged < 50 years (MI < 50), patients with MI aged MI ≥50 years (MI ≥50) and and young people without history of MI/CAD aged < 50 years (no-MI < 50)

	MI < 50 n = 240	MI ≥50 n = 240	no-MI < 50 n = 240	p value	p value
	a	b	c	a vs b	a vs c
Family history of MI/stroke in the first-degree relatives – all; n (%)	79 (32.9)	23 (9.6)	28 (11.7)	< 0.0001	< 0.0001
Family history of MI/stroke in the first-degree relatives – with ≥2 affected; n (%)	9 (3.7)	1 (0.4)	3 (1.2)	< 0.0001	< 0.0001
Family history of MI/stroke in the first- and the second- degree relatives – all; n (%)	84 (35.9)	36 (15.6)	34 (14.2)	< 0.0001	< 0.0001
Family history of MI/stroke in the first- and the second-degree relatives – with ≥2 affected; n (%)	26 (10.8)	7 (2.9)	9 (3.7)	< 0.0001	< 0.0001

**Fig. 1:**
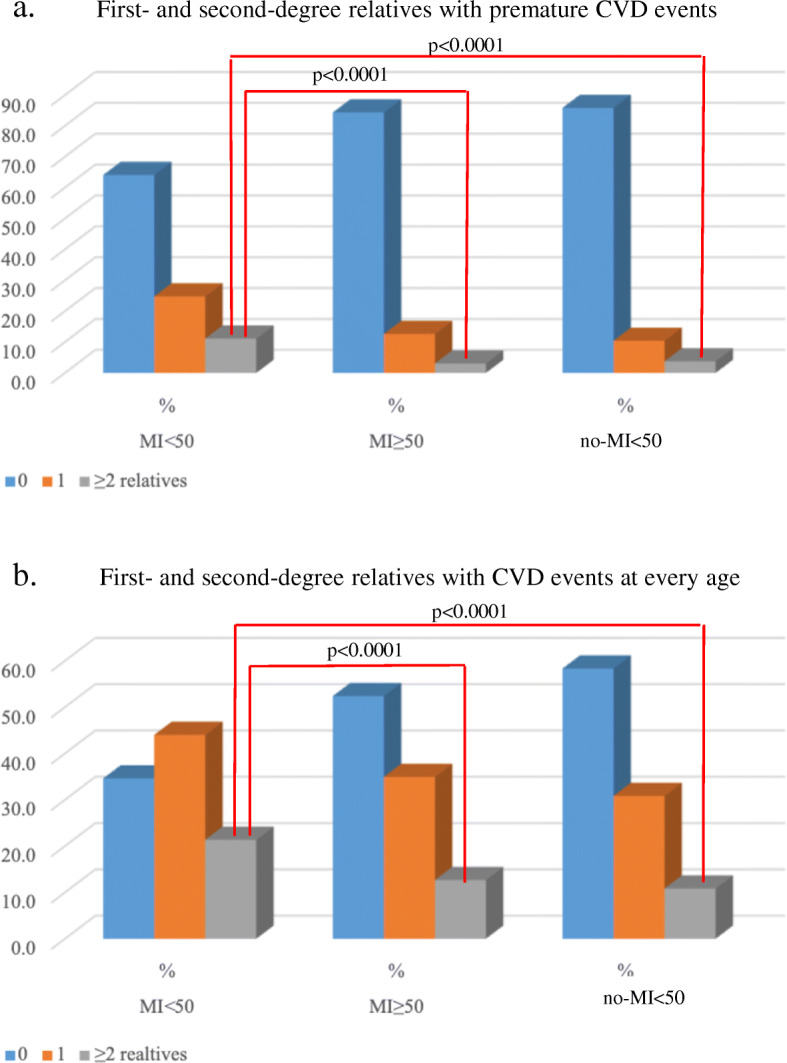
Differences among the MI < 50, MI ≥ 50 and no-MI < 50 groups in the percentage of cases with 0, 1 or ≥ 2 first- and second-degree relatives with a history of CVD events: **a** family history of premature CVD events with ≥2 affected relatives: 10.8% cases in the MI < 50 group vs 2.9% cases in the MI ≥50 group (*p* < 0.0001) and 10.8% cases in the MI < 50 group vs 3.7% cases in the no-MI < 50 group (p < 0.0001); **b** family history of CVD events with ≥2 affected relatives at every age: 21.4% cases in the MI < 50 group vs 12.7% cases in the MI ≥50 group (p < 0.0001) and 21.4% cases in the MI < 50 group vs 10.8% cases in the no-MI < 50 group (p < 0.0001)

The statistically significant differences among the MI < 50, MI ≥ 50 and no-MI < 50 groups also included the percentage of patients with ≥2 affected relatives, including parents, children, siblings, siblings of parents, and grandparents, with a history of premature CVD events: 10.8% vs 2.9% (*p* < 0.0001) and 10.8% vs 3.7% (p < 0.0001), respectively; Fig. 1.

**Fig. 2:**
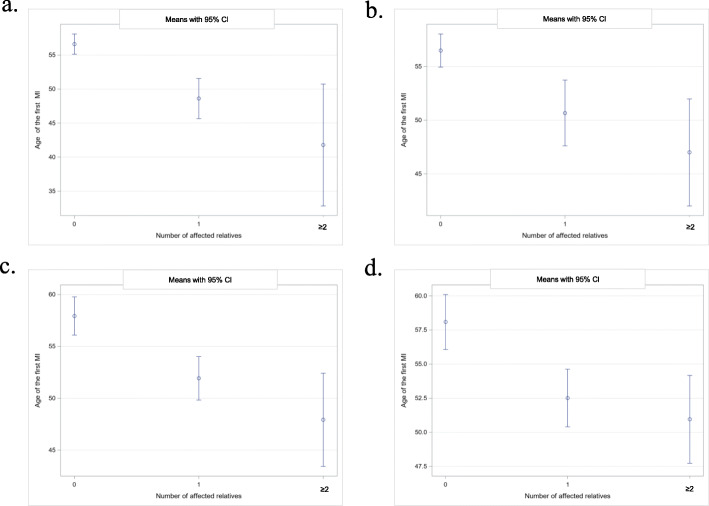
Relationship between the age of the patient’s first MI and the number of relatives with a history of CVD events. **a** Mean age with 95% Confidence Interval (CI) of patients with first episode MI: without a family history of premature CVD in first-degree relatives, with 1 affected relative and with ≥2 affected relatives: 56.6 [55.1–58.1], 48.6 [45.7–51.6] and 41.8 [32.8–50.8] years, respectively. **b** Mean age with 95% CI of patients with first episode MI: without a family history of premature CVD in first- and second-degree relatives, with 1 affected relative and with ≥2 affected first-degree relatives: 56.5 [55.0–58.0], 50.7 [47.6–53,7] and 47.0 [42.0–52.0] years, respectively. **c** Mean age with 95% CI of patients with first episode MI: without a family history of CVD at any age in first-degree relatives, with 1 affected relative and with ≥2 affected relatives: 57.9 [56.1–59.8], 51.9 [49.8–54.0] and 47.9 [43.4–52.4] years, respectively. **d** Mean age with 95% CI of patients with first episode MI: without a family history of CVD at any age in first- and second-degree relatives, with 1 affected relative and with ≥2 affected relatives: 58.1 [56.1–60.1], 52.5 [50.4–54.6] and 50.9 [47.7–54.2] years, respectively

There were statistically significant differences in the age of the first episode of MI between patients without a family history of premature CVD and patients with 1 affected relative (56.6 vs 48.6 years, respectively, *p* < 0.0001) and with ≥2 affected first-degree relatives (56.6 vs 41.8 years, respectively, *p* < 0.0001); Table 4. These differences were also significant for first- and second-degree relatives with premature CVD: 56.5 years for patients without affected relatives vs 50.7 for patients with 1 affected relative (*p* = 0.0003) and vs 47.0 years for patients with ≥2 affected relatives (*p* < 0.0001). There were also statistically significant differences in the age of the first episode of MI among patients without a family history of CVD at any age and patients with 1 affected relative (57.9 vs 51.9 years, respectively, p < 0.0001) and with ≥2 affected first-degree relatives (57.9 vs 47.9 years, respectively; p < 0.0001). These differences were also significant for first- and second-degree relatives with CVD event at any age: 58.1 years for patients without affected relatives vs 52.5 for patients with 1 affected relative (*p* = 0.0002) and vs 50.9 years for patients with ≥2 affected relatives (*p* = 0.0005). There was a clear reversal association between the age of the first episode MI and the number of first-degree relatives with a history of premature MI/stroke (Fig. 2).

**Table 4:** Differences in the age of the first episode MI among patients with 0, 1 and ≥ 2 affected relatives with the history of premature CVD events and CVD events at any age

Number of relatives:	0	1	≥2	0 vs 1 Delta [95% CI]	0 vs ≥2 Delta [95% CI]
Family history of premature CVD events					
Patients with first-degree relatives affected years ±SD (n)	56.6 ± 15.3 (*n* = 363)	48.6 ± 11.1 (*n* = 92)	41.8 ± 5.9 (*n* = 10)	8.0 [4.0–12.0]	14.8 [4.0–25.7]
*p*				*< 0.0001*	*< 0.0001*
Patients with first- and second degree relatives affected years ±SD (n)	56.5 ± 15.3 (*n* = 344)	50.7 ± 12.6 (*n* = 87)	47.0 ± 10.9 (*n* = 32)	5.8 [1.7–9.9]	9.5 [3.2–15.7]
*p*				*0.0003*	*< 0.0001*
Family history of CVD events at every age					
Patients with first-degree relatives affected years ±SD (n)	57.9 ± 15.7 (*n* = 239)	51.9 ± 13.5 (*n* = 184)	47.9 ± 10.1 (n-40)	6.0 [2.7–9.3]	10.0 [4.2–15.8]
*p*				*< 0.0001*	*< 0.0001*
Patients with first- and second degree relatives affected years ±SD (n)	58.1 ± 15.7 (*n* = 201)	52.5 ± 13.5 (*n* = 183)	50.9 ± 13.6 (*n* = 79)	5.6 [2.1–9.1]	7.1 [2.6–11.7]
*p*				*0.0002*	*0.0005*

When only MI, without ischemic stroke, was included in the family history, there were similar statistically significant differences in the age of the first episode MI among patients without a family history of MI, premature and at any age as well, with 1 affected relative and with ≥2 affected relatives (Table in Supplementary Materials). There was also a significant reversal association between the age of the first episode MI and the number of affected relatives, including first-degree and first- and second degree relatives with a history of premature MI and MI at any age as well.

## Discussion

In our study, there was a significantly higher incidence of a family history of premature CVD events in patients with MI at age < 50 in comparison to patients with MI at age ≥ 50 and to young people without history of MI/CAD. A family history of early MI or stroke is a widely recognized risk factor for MI at a young age. In the Malmo Diet and Cancer Study, a family history of coronary heart disease (CHD) was associated with an incidence of CHD with a hazard ratio of 1.52 (95% CI: 1.39–1.65), and only a small proportion of the family history effect was mediated by hypertension, hyperlipidaemia and diabetes [15].

Although the highest cardiovascular risk was associated with a maternal history at age < 50 years and a paternal history at age < 55 years, no substantial differences were seen between maternal and paternal positive CVD history [16]. In a Dutch cohort study, a particularly high incidence of CVD was revealed in people with parental onset of MI before age 70, with maternal history of MI before age 60 being the strongest predictor of CVD incidence [17].The offspring age of onset of CVD is significantly associated with both maternal and paternal age of CVD onset [18]. Nevertheless, data regarding the role of a family history of CVD that includes relatives other than parents or the number of affected family members are scarce.

In our study, there were significant differences among the MI < 50, MI ≥ 50 and no-MI < 50 groups in positive family history of CVD, and these differences involved not only the prevalence of premature CVD events restricted to parents but also such events in other first- and second-degree relatives. Moreover, there were statistically significant differences among the studied groups in the prevalence of CVD events at every age in family members (the first- and the second-degree relatives). A higher number of relatives with a positive history of CAD, including parents, children, siblings, siblings of parents and grandparents, was associated with a younger age of MI. The Danish Nationwide Cohort Study revealed that a history of MI in a combination of first- and second-degree relatives increased risks 1.8- to 7-fold in middle-aged persons [19]. Moreover, it was shown in this study that younger age at MI in relatives was associated with higher MI risk.

Interestingly, there was a statistically significant reversal association between the age of the first episode of MI in our patients and the number of relatives with a history of premature MI/stroke, and this relationship was particularly evident in the analysis involving the first-degree relatives, but not exclusively. An Italian study revealed that being a relative (including parents, siblings and siblings of parents) of an early-onset MI patient confers an adjusted hazard ratio of 2.7 for such events [20]. There are also data indicating that early-onset hypertension in grandparents raises the risk for hypertension in grandchildren, even after adjusting for early-onset hypertension in parents and for lifestyle factors [21].

Among other risk factors, the prevalence of smoking, hypertension, DM as well as BMI, HDL, TG and glucose levels differ significantly between the MI < 50 group and both control groups (MI ≥ 50 and no-MI < 50) in our study. Such findings are independent of region and patient ethnicity across the literature [22, 23]. For instance, our data are in concordance with recently published data from New Zealand conducted in a more complex population, including Caucasians, Maori and Pacific islanders [24]. Although smoking, hyperlipidaemia and obesity are crucial among the risk factors for MI at a young age, there are some differences in their distribution between particular groups of patients [25, 26]. For example, the strongest predictor of ACS in women ≤45 years of age was diabetes, with a 6-fold increase in risk [27]. Our study confirmed the significance of smoking, dyslipidaemia, obesity and carbohydrate metabolism disturbances as CAD risk factors.

The major limitation of our study was the relatively small number of patients; thus, the findings are difficult to apply to a larger, more diverse population. On the other hand, the high homogeneity of the groups, limited to the Polish population of Caucasian ethnicity, could be of value regarding the potential population and racial differences in the pathogenesis of CAD, particularly taking into account heritable risk factors. The control group of young healthy blood donors, usually more educated and more conscious of lifestyle than the general population, may not represent the community at large. Thus, the data coming from this group, including the prevalence of smoking, hypertension, DM, lipids levels or BMI may not be representative of the general population. On the other hand, the fact that donors stayed free from CAD until the age of 50, whereas our young patients suffered from MI before this age, enhances the role of lifestyle in CAD prevention.

## Conclusions

This study revealed that a younger age of MI is associated with a higher number of relatives with a history of premature atherosclerosis. A family history of premature atherosclerosis involving not only first- but also second-degree relatives seems to be a valuable factor in CAD risk evaluation in young people. The utility of these findings seems to be important for individual lifestyle change interventions as rational CVD prevention. Once a positive family history, particularly strengthened by such data, has been established, the health-care provider can emphasize the increased likelihood of MI at a young age as a strong incentive for patient-dedicated improvement in adherence to healthy lifestyles and medical regimens.

Family history of CVD is an effective indicator that one will have or develop key cardiovascular risk factors. Further analyses are required to determine whether a family history of premature CVD is associated with an increased likelihood of ACS at a young age independent of traditional cardiovascular risk factors.

## Supplementary information

**Additional file 1: Table 1.** Differences in the age of the first episode MI among patients with 0, 1 and >=2 affected relatives with the history of premature MI and MI at any age.

## Data Availability

The datasets used and/or analysed during the current study are available from the corresponding author on reasonable request.

## Abbreviations

ACS: Acute coronary syndrome
ADA: American Diabetes Association
AHA: American Heart Association
BMI: Body mass index
CAC: Coronary artery calcification
CAD: Coronary artery disease
CHD: Coronary heart disease
CVD: Cardiovascular disease
DBP: Diastolic blood pressure
HDL: High-density lipoprotein
LDL: Low-density lipoprotein
MI: Myocardial infarction
NSTEMI: Non-ST-elevation MI
SBP: Systolic blood pressure
STEMI: ST-elevation MI
TG: Triglyceride
UA: Unstable angina
WHO: World Health Organization
