# Supplementary material for: Younger age of patients with myocardial infarction is associated with a higher number of relatives with a history of premature atherosclerosis
Source: BMC Cardiovasc Disord. 2020 Sep 11;20:410. doi: 10.1186/s12872-020-01677-w (PMC7488448; doi:10.1186/s12872-020-01677-w)
Supplement: Supplementary file 1 — Additional file 1: Table 1. Differences in the age of the first episode MI among patients with 0, 1 and >=2 affected relatives with the history of premature MI and MI at any age. [file 12872_2020_1677_MOESM1_ESM.doc]

Table 1

Differences in the age of the first episode MI among patients with 0, 1 and >2 affected relatives with the history of premature MI and MI at any age.

| Number of relatives: | 0 | 1 | >2 | 0 vs 1  Delta [95% CI] | 0 vs >2  Delta [95% CI] |
| --- | --- | --- | --- | --- | --- |
| Family history of premature MI | | | | | |
| Patients with first-degree relatives affected  years ±SD (n) | 56.4 ± 15.3  (n=373) | 48.5 ± 10.8  (n=83) | 41.7 ± 6.2  (n=9) | 7.9 [3.7-12.0] | 14.7 [3.3-26.2] |
| *p* |  |  |  | *<0.0001* | *<0.0001* |
| Patients with first- and second degree relatives affected  years ±SD (n) | 56.2 ± 15.2  (n=354) | 51.1 ± 13.4  (n=80) | 46.7 ± 11.1  (n=30) | 5.1 [0.8-9.3] | 9.5 [3.0-16.1] |
| *p* |  |  |  | *0.006* | *<0.0001* |
| Family history of MI at every age | | | | | |
| Patients with first-degree relatives affected  years ±SD (n) | 57.5 ± 15.6  (n=261) | 51.7 ± 13.4  (n=167) | 48.2 ± 10.7  (n=35) | 5.8 [2.4-9.1] | 9.3 [3.1-15.4] |
| *p* |  |  |  | *<0.0001* | *<0.0001* |
| Patients with first- and second degree relatives affected  years ±SD (n) | 57.8 ± 15.6  (n=225) | 51.9 ± 13.2  (n=167) | 51.2 ± 14.2  (n=71) | 5.9 [2.4-9.4] | 6.6 [2.0-11.3] |
| *p* |  |  |  | *<0.0001* | *0.0016* |
